# Supplementary material for: Loss of Otopetrin 1 affects thermoregulation during fasting in mice
Source: PLoS One. 2023 Oct 9;18(10):e0292610. doi: 10.1371/journal.pone.0292610 (PMC10561838; doi:10.1371/journal.pone.0292610)
Supplement: S1 Fig — BAT mRNA from 3 male mice of each genotype was studied. Mapped reads from RNA-Seq in exon 1 of the Otop1 gene, with the site of the 38-bp deletion indicated with a box. Black: WT; Red: Otop1-/-. Each row is a different mouse. (PDF) [file pone.0292610.s001.pdf]

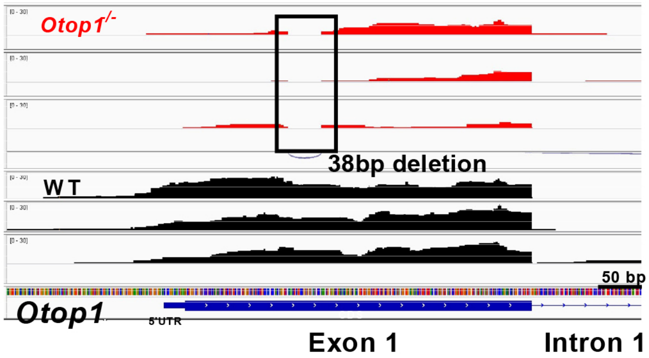

Supplementary Figure 1. RNA-Seq showed 38bp deletion in exon 1 of *Otop1* in BAT of *Otop1*$^{-/-}$ mice. BAT mRNA from 3 male mice of each genotype was studied. Mapped reads from RNA-Seq in exon 1 of the *Otop1* gene, with the site of the 38-bp deletion indicated with a box. Black: WT; Red: *Otop1*$^{-/-}$. Each row is a different mouse.
