# Supplementary material for: Loss of Otopetrin 1 affects thermoregulation during fasting in mice
Source: PLoS One. 2023 Oct 9;18(10):e0292610. doi: 10.1371/journal.pone.0292610 (PMC10561838; doi:10.1371/journal.pone.0292610)
Supplement: S2 Fig — (A) Volcano plot of BAT RNA-Seq results. Gene names are indicated for the 10 most significantly different RNAs between wild type and Otop1-/- mice. BAT mRNA level by qPCR in chow-fed WT and Otop1-/- male mice (aged 17–19 weeks) for (B) differentially expressed genes identified by RNA-Seq and (C) known BAT genes. n = 6/group; p-value from unpaired t-test. (D) BAT mRNA level by qPCR in HFD-fed WT and Otop1-/- male mice (aged 45 weeks) for known BAT genes. n = 6/group; p-value from unpaired t-test. mRNA levels were normalized to Tbp gene. (PDF) [file pone.0292610.s002.pdf]

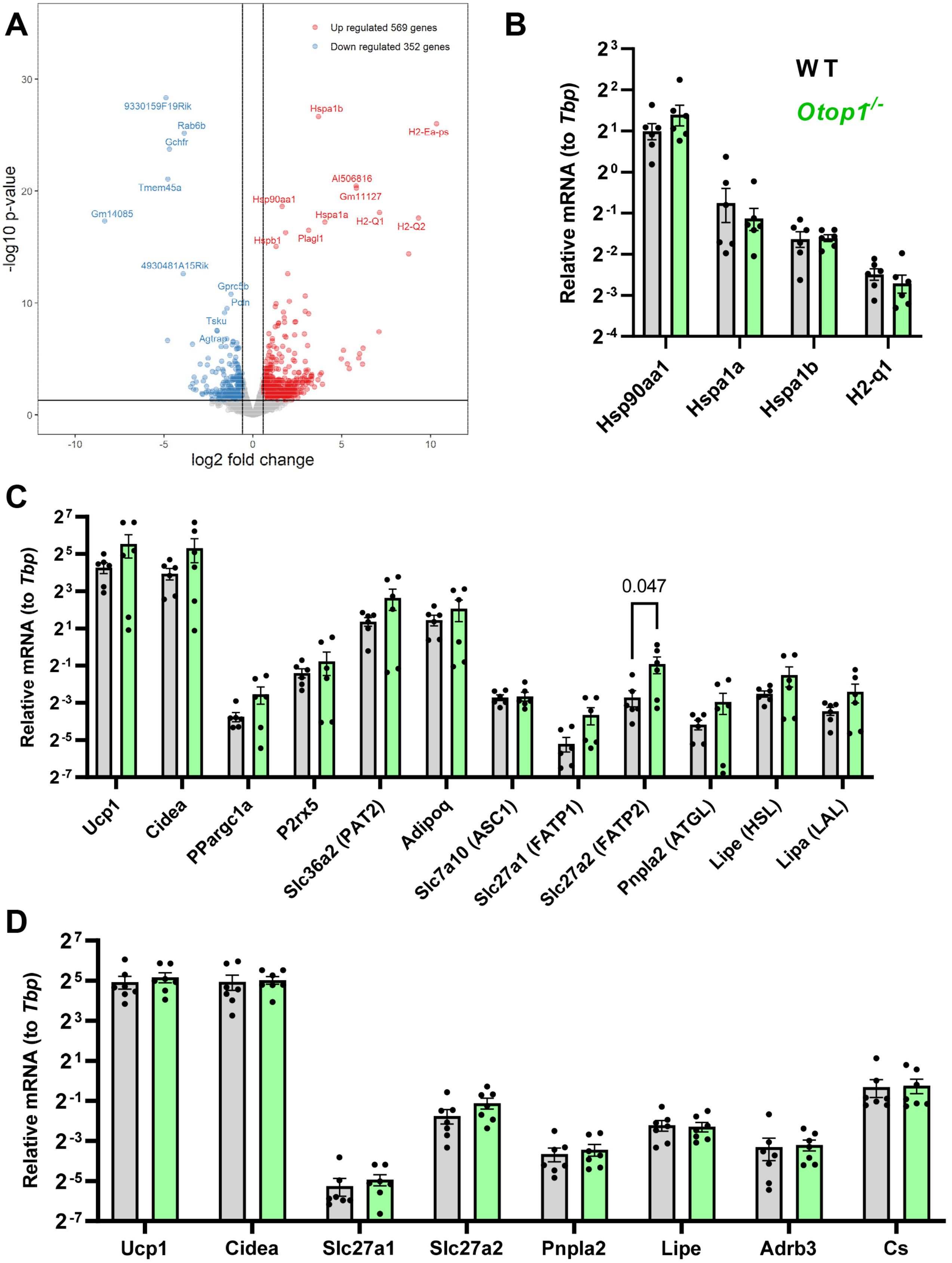

Supplementary Figure 2. Gene expression of BAT mRNA by qPCR. (A) Volcano plot of BAT RNA-Seq results. Gene names are indicated for the 10 most significantly different RNAs between wild type and *Otop1*$^{-/-}$ mice. BAT mRNA level by qPCR in chow-fed WT and *Otop1*$^{-/-}$ male mice (aged 17-19 weeks) for (B) differentially expressed genes identified by RNA-Seq and (C) known BAT genes. n=6/group; p-value from unpaired t-test. (D) BAT mRNA level by qPCR in HFD-fed WT and *Otop1*$^{-/-}$ male mice (aged 45 weeks) for known BAT genes. n=6/group; p-value from unpaired t-test. mRNA levels were normalized to *Tbp* gene.
