# Supplementary material for: Loss of Otopetrin 1 affects thermoregulation during fasting in mice
Source: PLoS One. 2023 Oct 9;18(10):e0292610. doi: 10.1371/journal.pone.0292610 (PMC10561838; doi:10.1371/journal.pone.0292610)
Supplement: S3 Fig — BAT mRNA from male mice measured by qPCR (Black: WT; Green; Otop1-/-; n = 6–7; p-value calculated by t-test). (PDF) [file pone.0292610.s003.pdf]

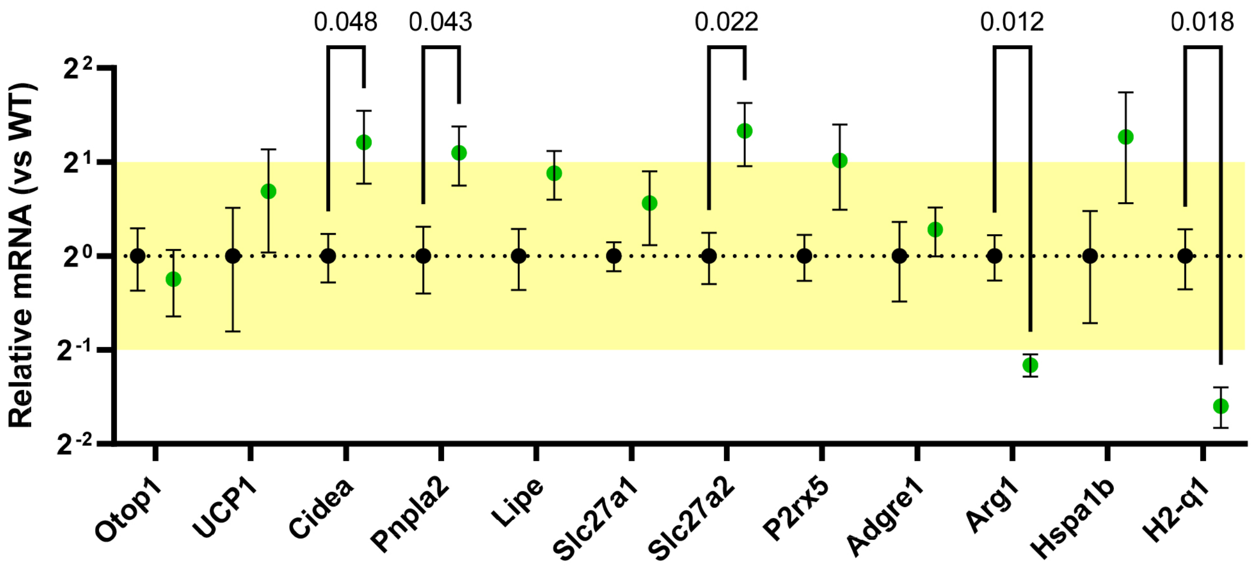

Supplementary Figure 3. Gene expression in BAT from WT and *Otop1*$^{-/-}$ mice after a 24-hour fast. BAT mRNA from male mice measured by qPCR (Black: WT; Green; *Otop1*$^{-/-}$; n=6-7; p-value calculated by t-test)
