# Supplementary material for: Loss of Otopetrin 1 affects thermoregulation during fasting in mice
Source: PLoS One. 2023 Oct 9;18(10):e0292610. doi: 10.1371/journal.pone.0292610 (PMC10561838; doi:10.1371/journal.pone.0292610)
Supplement: S4 Fig — (A) OCR ratio (normalized to basal OCR) in response to CL316243. (B) CL:Basal OCR ratio is the mean of 6 measurements after CL316243 application. n = 6/group; p-value from unpaired t-test. (C) OCR/ECAR ratio. n = 6/group. (PDF) [file pone.0292610.s004.pdf]

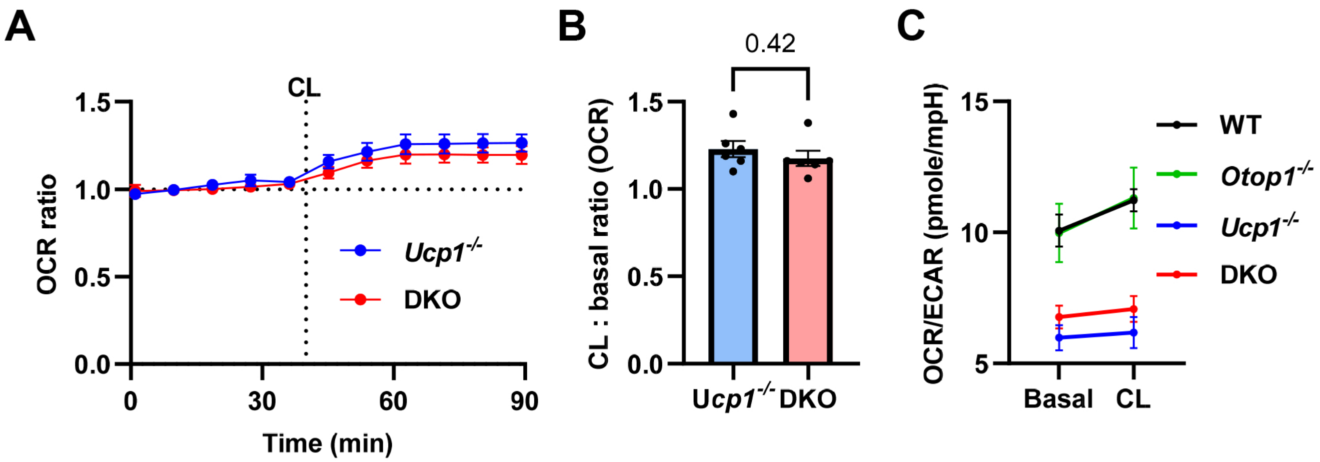

Supplementary Figure 4. Loss of *Ucp1* decreases basal oxygen consumption rate (OCR) and response to CL316243 in brown adipose tissue (BAT). (A) OCR ratio (normalized to basal OCR) in response to CL316243. (B) CL: Basal OCR ratio is the mean of 6 measurements after CL316243 application. n=6/group; p-value from unpaired t-test. (C) OCR/ECAR ratio. n=6/group.
