# Supplementary material for: Loss of Otopetrin 1 affects thermoregulation during fasting in mice
Source: PLoS One. 2023 Oct 9;18(10):e0292610. doi: 10.1371/journal.pone.0292610 (PMC10561838; doi:10.1371/journal.pone.0292610)
Supplement: S5 Fig — (A) Body temperature (Tb), (B) total energy expenditure (TEE), and (C) RER of male mice from the indicated genotypes, in response to CL316243 (0.1 mg/kg, ip., injected at time 0). The bar graphs show the mean at 0 to 5 hr. n = 5-6/group; p-values from 3-way ANOVA with Tukey post-hoc testing. (PDF) [file pone.0292610.s005.pdf]

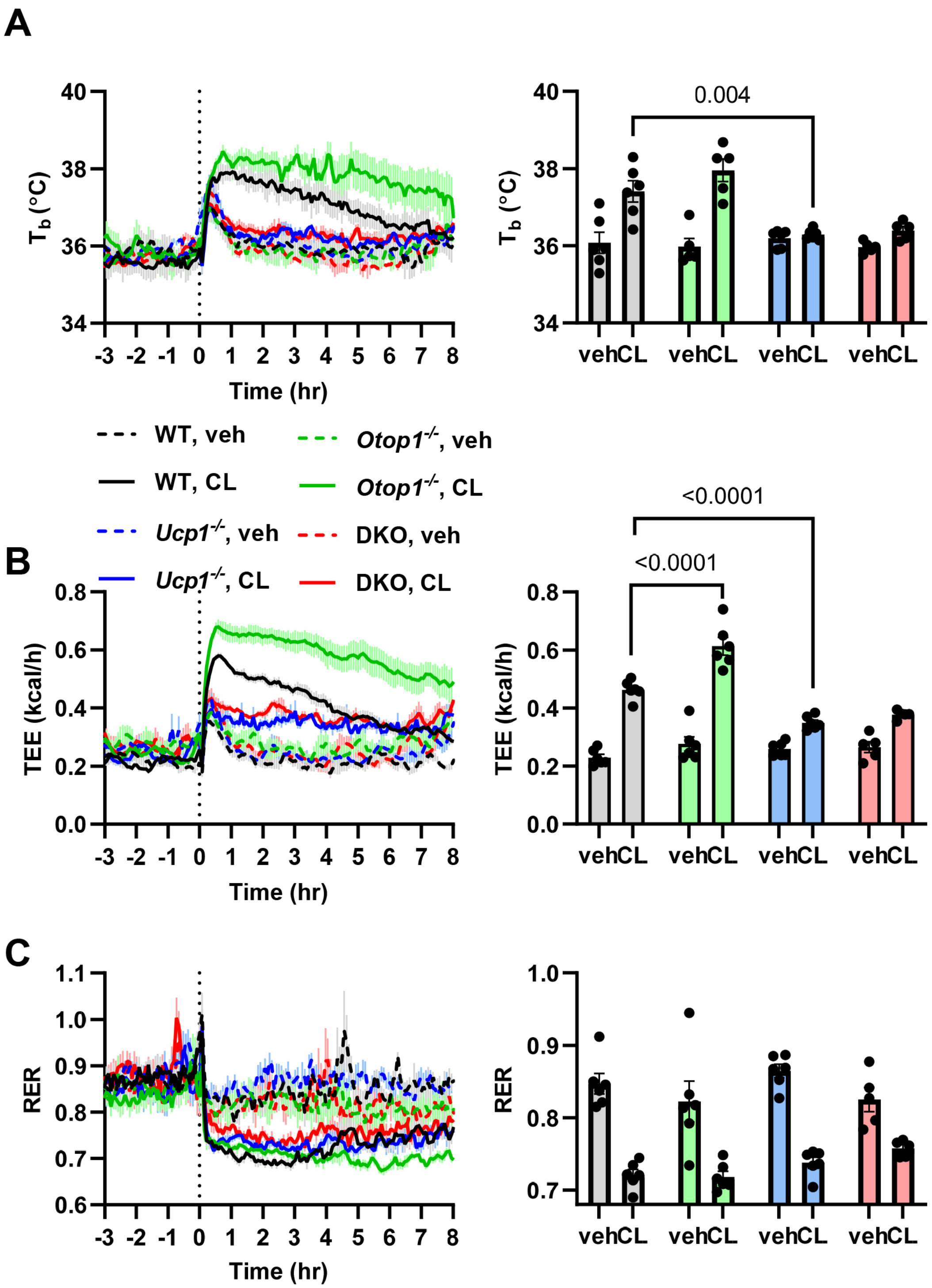

Supplementary Figure 5. Loss of *Ucp1* decreases body temperature and energy expenditure stimulated by β3-agonist. (A) Body temperature (T_b), (B) total energy expenditure (TEE), and (C) RER of male mice from the indicated genotypes, in response to CL316243 (0.1 mg/kg, ip., injected at time 0). The bar graphs shows the mean at 0 to 5 hr. n=5-6/group; p-values from 3-way ANOVA with Tukey post-hoc testing.
