# Supplementary figures and images for: Loss of Otopetrin 1 affects thermoregulation during fasting in mice
Source: PLoS One. 2023 Oct 9;18(10):e0292610. doi: 10.1371/journal.pone.0292610 (PMC10561838; doi:10.1371/journal.pone.0292610)

## Slide 1
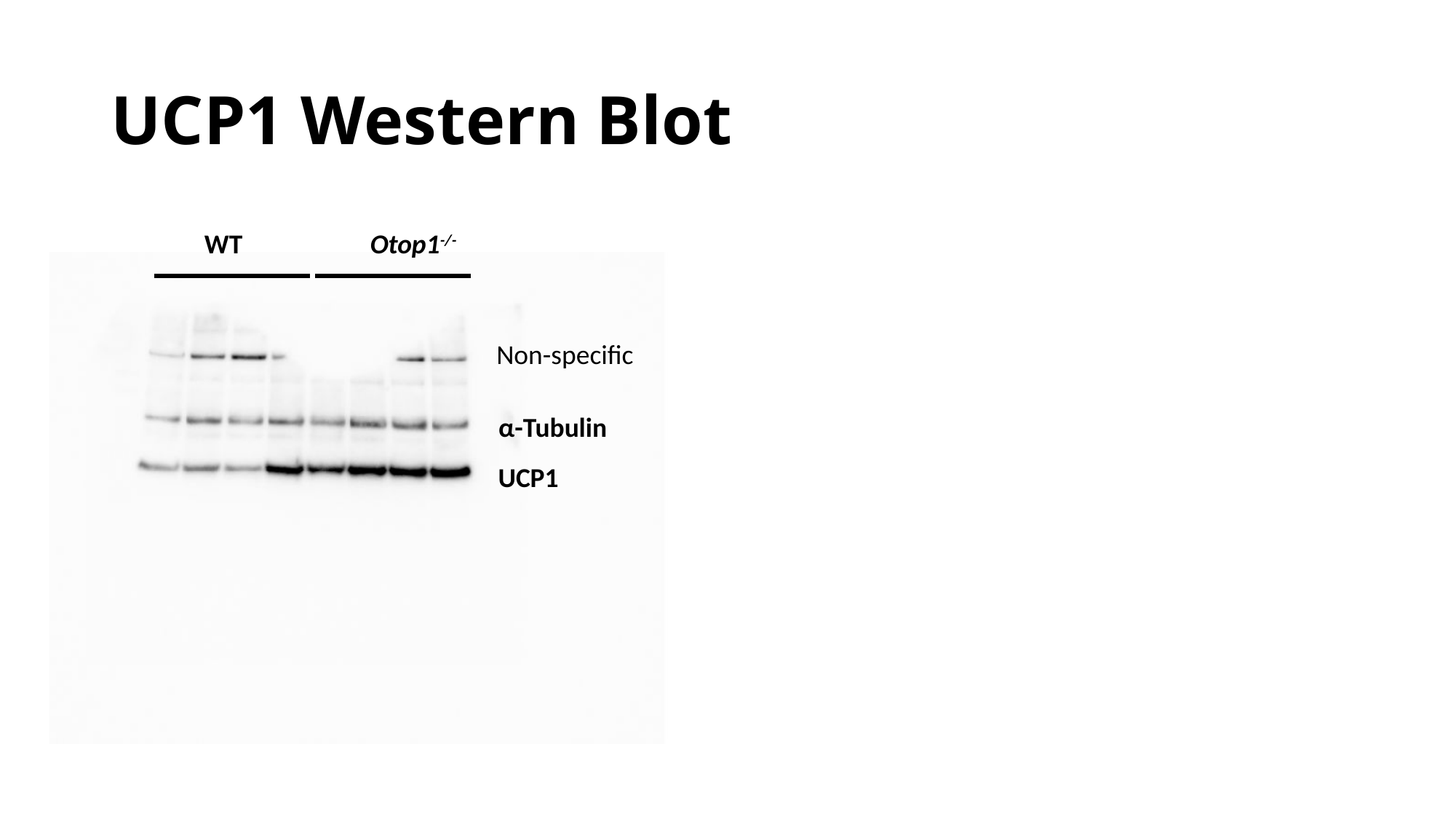

# UCP1 Western Blot
WT
Otop1-/-
Non-specific
α-Tubulin
UCP1

Supplement: S7 Fig — (PPTX) [file pone.0292610.s007.pptx]
