# Supplementary material for: Loss of Otopetrin 1 affects thermoregulation during fasting in mice
Source: PLoS One. 2023 Oct 9;18(10):e0292610. doi: 10.1371/journal.pone.0292610 (PMC10561838; doi:10.1371/journal.pone.0292610)
Supplement: S1 Table — (PDF) [file pone.0292610.s008.pdf]

Supplementary Table 1. PCR primer sets.

Gene Name	Forward primer	Reverse primer
Tbp	TTTGTGCCAGATACATTCCG	AACAATTTACAAGCTGCGTTT
Otop1	ACGGCCTTTCTGTTTCTTTGC	CCGTTGTCATACTCGGGACG
Ucp1	ACTGCCACACCTCCAGTCATT	CTTTGCCTCACTCAGGATTGG
Cidea	TGCTCTTCTGTATCGCCCAGT	GCCGTGTTAAGGAATCTGCTG
Ppargc1a (PGC-1α)	AGCCGTGACCACTGACAACGAG	GCTGCATGGTTCTGAGTGCTAAG
P2rx5	TTTGTCATTCCGTCTCAGGG	CCTCTGAACACTCGCCATCC
Pat2	AGAAGACCAAGGGCATAACCG	TTACCAGCAAGCTGAGTGGG
Adipoq	GCACTGGCAAGTTCTACTGCAA	GTAGGTGAAGAGAACGGCCTTGT
Slc7a10 (Asc1)	GTGCTTCAGCCTGTCTTTCCC	GTTCACCCACGTCAGGAGC
Slc27a1 (Fatp1)	AGCTCAGAACTTCCCAGTCC	GGTGGAGAGAGAGATCCCTGG
Slc27a2 (Fatp2)	AGAAGTCGCTGACATCGTGG	GAGGCCATCCCAATTCGACC
Pnpla2 (Atgl)	ACCTTCGCAATCTCTACCGC	CTCCAGCAGGGCATTCCTCC
Lipe (Hsl)	GCAAAGAAGGATCGAAGAACC	GTGTCATCGTGCGTAAATCC
Lipa (Lal)	AGGTTGGAGGACCACTCCC	CTGATACAGTTCCCGTGGGC
Lgals3 (Mac-2)	CAGAGAGCACTACCCAGGAAAAT	TGAGGGTTTGGGTTTCCAGAG
Arg1	ACACGGCAGTGGCTTTAACC	TGGCGCATTCACAGTCACTT
Hsp90aa1	TTGCGGTACTACACATCTGC	GTTAGCAACCTGGTCCTTGG
Hspa1a	AGTTTGTCCTGCAATCAAGTCC	ACAGGGAAGATAAAGCCCACG
Hspa1b	TCGAGGAGGTGGATTAGAGGC	ACCTTGACAGTAATCGGTGCC
H2-q1	ACTACAACCAGAGCAAGGGC	GCAGGTACCCGCTTTGAAGG
Pparg (Ppar-γ)	GCATGGTGCCTTCGCTGA	TGGCATCTCTGTGTCAACCATG
Srebf1	AAGCAAATCACTGAAGGACCTGG	AAAGACAAGGGGCTACTCTGGGAG
Ehhadh	GGCTGAGTATCTGAGGCTGC	TGGCTCTAACCGTATGGTCC
Fasn	AAGTTGCCCGAGTCAGAGAACC	ATCCATAGAGCCCAGCCTTCCATC
Adgre1 (F4/80)	CCAGGCTTTGTCTTGAATGGC	AGCTTCCGAGAGTGTTGTGG
Oasl1	CTTCACGATCCAGACCAGGG	TCAGATTTGCGTAGACCTCGG
Oasl2	TCTGTTGCACGACTGTAGGC	TGTCCAATCCACTGTTCCCG
Cd68	CTGCTCTCTCTAAGGCTACAGG	AGCAAGAGGGACTGGTCACG
Ifi44	ACATCTTAAAGGGCCACACTCC	CCTTCAGCAGTGGGTCATGG
Ifitm3	GGATTCCGACTTCCGGTCC	TGTTACACCTGCGTGTAGGG
Ifih1	GATGTTCTGCGCCAAACTGG	TCGAAGCAGCTGACACTTCC
Ccl2	TCAGCCAGATGCAGTTAACGC	TCTGGACCCATTCCTTCTTGG
Ifng (Ifn-γ)	TAGCCAAGACTGTGATTGCGG	AGACATCTCCTCCCATCAGCAG
Tnf (Tnf-α)	AAGCCTGTAGCCCACGTCGTA	AGGTACAACCCATCGGCTGG
