# Supplementary material for: Loss of Otopetrin 1 affects thermoregulation during fasting in mice
Source: PLoS One. 2023 Oct 9;18(10):e0292610. doi: 10.1371/journal.pone.0292610 (PMC10561838; doi:10.1371/journal.pone.0292610)
Supplement: S6 Fig — (A-I) Plasma metabolite levels in littermate male chow- and HFD-fed mice. (J,K) Insulin tolerance test (0.75 u/kg) in chow and HFD mice. (L-N) Glucose tolerance test in chow (2 g/kg) and HFD (1 g/kg) mice. Chow mice were studied at ~23 weeks (mean body weights: WT, 24.5 g; Otop1-/- 23.3 g). HFD mice were studied at ~34 weeks, after 8 weeks on the HFD diet (mean body weights: WT, 35.1 g; Otop1-/- 30.2 g; p = 0.13). n = 4-7/group, p-values from unpaired t-test. (PDF) [file pone.0292610.s006.pdf]

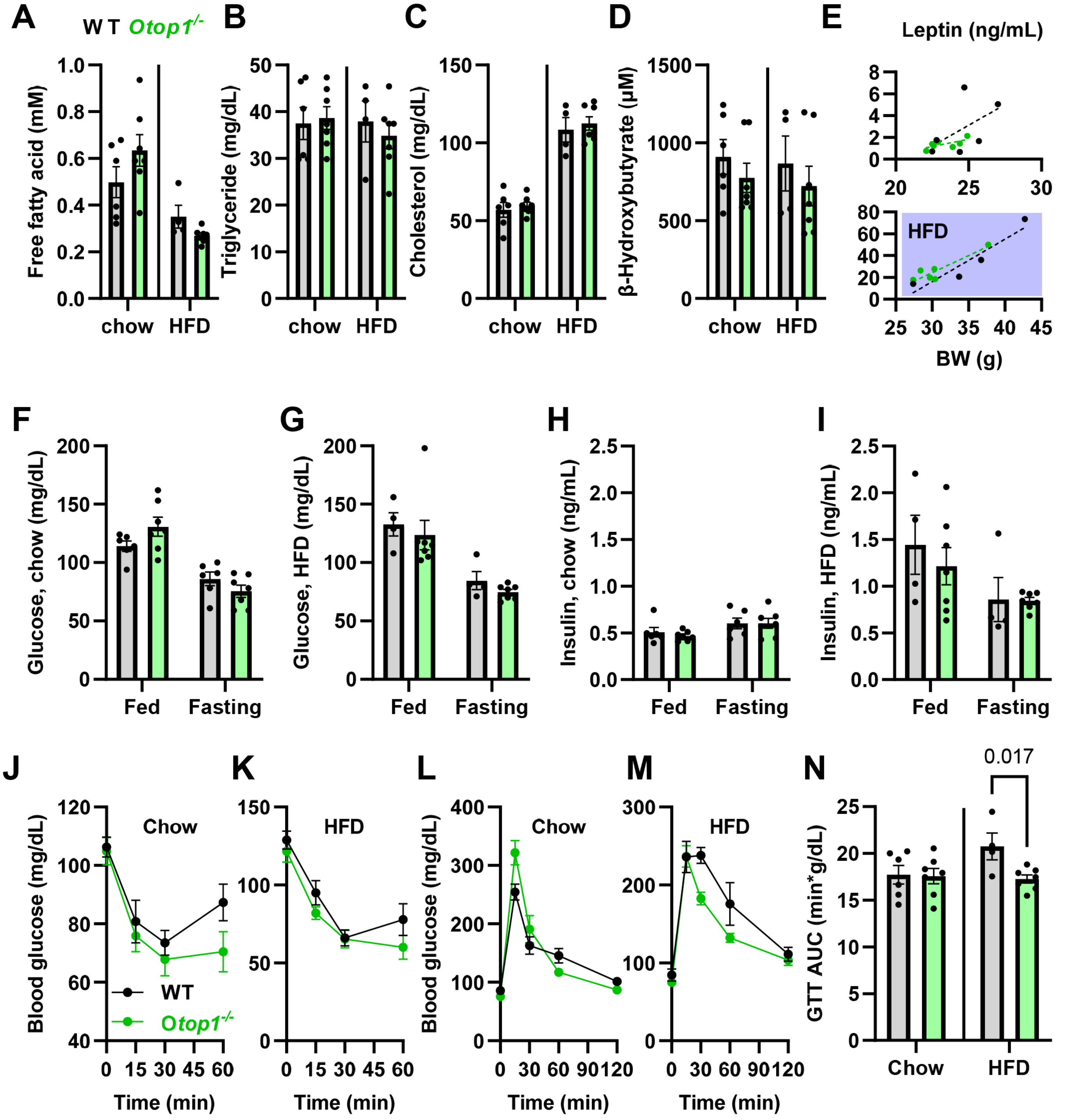

Supplementary Figure 6. Metabolic parameters in *Otop1*$^{-/-}$ female mice. (A-I) Plasma metabolite levels in littermate male chow- and HFD-fed mice. (J,K) Insulin tolerance test (0.75 u/kg) in chow and HFD mice. (L-N) Glucose tolerance test in chow (2 g/kg) and HFD (1 g/kg) mice. Chow mice were studied at ~23 weeks (mean body weights: WT, 24.5 g; *Otop1*$^{-/-}$ 23.3 g). HFD mice were studied at ~34 weeks, after 8 weeks on the HFD diet (mean body weights: WT, 35.1 g; *Otop1*$^{-/-}$ 30.2 g; p=0.13). n=4-7/group, p-values from unpaired t-test.
