# Supplementary material for: Loss of Otopetrin 1 affects thermoregulation during fasting in mice
Source: PLoS One. 2023 Oct 9;18(10):e0292610. doi: 10.1371/journal.pone.0292610 (PMC10561838; doi:10.1371/journal.pone.0292610)
Supplement: Attachment — Submitted filename: response to reviewers 2023.05.02.docx [file pone.0292610.s010.docx]

**RE:** Manuscript Number: PONE-D-22-34443

**Title:** "Physiology of mice lacking Otopetrin 1 (OTOP1) function"

May 2, 2023

Dear Dr Qiao and reviewers,

We thank the reviewers for their thoughtful and useful comments and invitation to submit a revised manuscript. We have addressed all of the reviewer comments and suggestions, which have improved the manuscript. The detailed answers are below (reviewer’s comments in black and our responses in red) and significant changes are shown in yellow in a supplemental file.

As a general comment, we now make clearer that the phenotype changes caused by the loss of OTOP1 function are mild. For example, this includes changing the ms title to “Loss of Otopetrin 1 affects thermoregulation during fasting in mice”. We have revised and condensed the discussion of how the loss of proton flux might explain the subtle changes and removed the associated supplementary figure.

Regards,

Marc

Reviewer #1: OTOP1 is a proton channel that is highly expressed in BAT. In the present study, Tu et al. found that Otop1-/- mice had similar body temperature as control mice at baseline and in response to cold and hot temperature. Interestingly, Otop1-/- mice exhibited an exaggerated hypothermia in response to fasting. Oxygen consumption upon stimulation with CL316243 (b3-adrenergic agonist) was significantly increased. A previous study reported that OTOP1 mutant mice exhibit insulin resistance, but the insulin sensitivity was normal in this study. The authors concluded that loss of OTOP1 does not change basal function of BAT, but affects stimulated responses. The findings are potentially interesting, but there are several questions that deflate the value of the present study.

Major criticisms

Figure 1. Western blotting of OTOP1 is necessary.

As suggested, we attempted Western blotting for OTOP1 in BAT lysates. The GAPDH antibody (Sigma G8795) gave the expected 36 kDa band on SDS PAGE. However, commercial antibodies advertised as targeting OTOP1 antibodies gave Western bands of the following sizes, none of which match the 66 kDa expected for OTOP1: Novus NBP1-86306 53 kDa; Alomone AHC-005 59 kDa; Abnova H00133060-M02 59 kDa; MyBioSource MBS6003792, 80 kDa). Due to this variability, we are not confident in the specificity of any of these antibodies and will not include OTOP1 Westerns in the paper. We added a sentence to the Results (line 167-169) “We were unsuccessful in measuring OTOP1 protein levels by Western blotting of BAT since none of the four commercial antibodies putatively to OTOP1 detected a band of the correct size in wild type mice (see Methods)”. We also added section to the Methods (lines 128-137).

“**2.6 Western blot analysis** BAT lysates from WT and Otop1-/- mice were prepared, separated using reducing SDS PAGE gels (Bolt Bis-Tris Plus mini-gel; ThermoFisher Scientific NW0412A), and transferred to Immobilon®-P PVDF Membrane (Millipore IPVH304F0). Primary antibodies targeting OTOP1 (1:500 dilution, Novus NBP1-86306; 1:500, Alomone AHC-005; 1:500, Abnova H00133060-M02; 1:500, MyBioSource MBS6003792), GAPDH (1:5000, Sigma G8795), UCP1 (1:1000, Sigma U6382), and α-tubulin (1:5000; Sigma T6074) were used. HRP-conjugated secondary antibodies (1:5000, Goat anti-Rabbit IgG, Thermofisher Scientific A16116; 1:5000, Goat anti-Mouse IgG, ThermoFisher Scientific 31430) were used with SuperSignal™ West Pico PLUS Chemiluminescent Substrate as recommended (ThermoFisher Scientific 34580).”

For the reviewers’ benefit, we include the anti-OTOP1 blots below. In the first three blots, the left four samples are WT and the right four are Otop1-/-. In the Novus antibody blot lanes 1,2,5,6 are WT and the others are Otop1-/-.

Abnova: Alomone:

MyBioSource: Novus:

Figure 2A and B. The authors described that “Otop1-/- male and female mice had the same body weights as controls”. However, it looks (especially female) that there is an attenuation of weight gain in the mutant mice. The authors started HFD feeding around 26-30 weeks of age. It is unusual (too late).

We have examined several cohorts for a body weight difference between WT and Otop1-/- mice. Three male Otop1-/- cohorts were studied. One chow cohort had insignificantly lower BW and another had a slightly higher BW that reached significance. In the third cohort (shown in Figure 2A) the BW was similar. One female cohort was studied, shown in Figure 2B. In the females on the HFD there was large variation in body weight, so the lower average Otop1-/- weight was not statistically significant. We modified the text in this section (line 180-183) “On a chow diet Otop1-/- male and female mice had similar body weights as controls (Figure 2A-B), also seen in two independent male cohorts (e.g., 28.0 g vs 31.7 g, wild type vs Otop1-/- at age 19 weeks, p=0.02; and 36.2 g vs 33.1 g, wild type vs Otop1-/- at age 38 weeks, p= 0.16; n=5-7/group). No difference was elicited by a high fat diet (HFD) to induce obesity (Figure 2A-B).”

Figure 3. The authors should show the UCP1 protein expression levels in this context. Palmitate affects OCR of the mutant mice. Why?
As suggested, we added a Western that shows no significant change in UCP1 protein levels in *Otop1^-/-^* mice (new Figure 1I). (line 172)

In the discussion we speculate that palmitate, as a weak acid, may provide a proton carrying function. (lines 303-305)

Figure 4. The body temperature of Otop1-/- mice in the fasting condition was significantly lower. Diet-induced thermogenesis may be impaired in the mutant mice. Decrease of physical activity at 23C might be also an important reason. What is the mechanism of reduced physical activity?

To address this we have added a reference and have modified the text (line 218-219) “Physical activity was reduced in the Otop1-/- mice at 23 ˚C, likely related to their vestibular deficit, since another mouse with a vestibular deficit also has decreased physical activity [24].”

How about the body temperature and physical activity of BAT specific Otop1 KO mice? More experiments and explanation are necessary.

We respectfully suggest that generation of a floxed Otop1 mouse and then study of a BAT specific Otop1 KO is beyond the scope of this ms.

The decrease of body temperature was not detected in the mutant mice fed a HFD. Why?

DIO mice have increased energy reserves, thus (line 225) “fasting is not as energetically challenging in obese mice.” Thus, the DIO control and Otop1-/- mice show the same hypothermia.

Figure 6. UCP1 plays an important role in the b3 agonist-induced oxygen consumption. Western blotting of UCP1 is required.

As suggested, we added a Ucp1 Western showing no significant change in UCP1 protein levels in *Otop1^-/-^* mice (new Figure 1I). (line 172)

Figure 7. They indicate that insulin sensitivity of the mutant mice is similar. This reviewer thinks that the sensitivity is better in the mutant (Figure 7K).
We re-analyzed the ITT data (Figure 7J,K). The t-test P values (WT vs Otop1-/-) comparing the ‘reverse AUC’, the AUC of the drop from the initial glucose value, were 0.24 and 0.66 for Figures 7J and 7K, respectively (the data for one WT mouse in Figure 7K contained an error, which is corrected in the updated Figure 7K).

Supplementary Figure 6 and discussion. The authors claimed that FCCP induced intracellular acidification and CL316243 (b3 agonist) induced intracellular alkalization. They propose that changes of pH cause proton influx/efflux. However, there are no direct experimental evidence to support the proposal.

In the Discussion, we cited the work of others to provide support for our hypothesis. To address the reviewer’s concerns, we have trimmed the Discussion and removed Supplementary Figure 6. The revised section is (lines 291-305)

“*Ex vivo*, *Otop1^-/-^* BAT basal mitochondrial respiration was unchanged, while maximal respiration was reduced and β3-adrenergic stimulated respiration was increased when compared to controls. Basally, taking into account the intracellular pH, extracellular pH, and plasma membrane potential, there is a small electrochemical gradient across the brown adipocyte plasma membrane that predicts inward flux of protons via OTOP1 [30, 31]. In the presence of FCCP, the plasma membrane is depolarized and the cytosol is acidified [32]; under these conditions OTOP1 could instead mediate proton efflux, thereby increasing intracellular pH. With FCCP, the lack of OTOP1 function might decrease the intracellular pH, potentially inhibiting processes including CPT1-mediated fatty acid transport [33] and glycolysis [34], thereby leading to the observed reduction in *Otop1^-/-^* OCR.

“Under stimulated conditions, such as with a β3-adrenergic agonist, BAT cytosol is alkalinized [31]. Cytoplasmic alkalinization could increase OTOP1-mediated proton influx across the plasma membrane. Thus, loss of *Otop1* would reduce that pH gradient across the inner mitochondrial membrane and thus cause a compensatory increase mitochondrial respiration to maintain the pH gradient. Additional palmitate could compensate for the loss of *Otop1,* as its protonated form might act as carrier to increase proton influx.”

Reviewer #2: This manuscript examined the thermogenic phenotype of Otopetrin 1 (Otop1)-deficient mice focusing on dissecting the role of Otop1 in brown adipose tissue. In general, systemic characterization of energy homeostasis by indirect calorimetry identified maintained body temperature at baseline and in response to cold and hot ambient temperature in Otop1-deficient mice, suggesting an indispensable role of Otop1 in BAT function despite its high expression. They did identify that Otop1-/- mice exhibited exaggerated hypothermia and hypometabolism under fasting without providing any explanation. They further analyzed BAT mitochondrial function by Seahorse analysis in the presence or absence of palmitate or after stimulation of CL316243. These data were rather confusing and failed to provide a clear picture of how exactly Otop1 loss exaggerates CL316243 stimulated response in BAT. The following concerns need to be addressed:
The manuscript title needs to be more specific: e.g. Otopetrin 1 is dispensable for thermoregulation in mice.

As suggested, we made the title more informative "Loss of Otopetrin 1 affects thermoregulation during fasting in mice”

Why there is only a 35% reduction of Otop1 mRNA in Otop1-/- mice? Western blot is needed to confirm the deletion of Otop1 in BAT, if possible other tissues in global Otop1-/- mice. Whether the low efficiency of Otop1 deletion explains the lack of similar metabolic phenotypes observed in HFD-fed Otop1tlt/tlt mice (ref 15). The discrepancy between these two mouse lines needs to be better discussed.

The Otop1-/- mice are a complete null for OTOP1 function. Physiologically, the Otop1-/- mice show head tilt and the inability to right themselves when swimming. Importantly, in electrophysiology studies the Otop1-/- mice have no detectable proton current in sour taste cells, while the Otop1 tlt/tlt mice have ~10% residual function [1].

Like the reviewer, we were surprised that the Otop1-/- deletion caused only 35% reduction in Otop1-/- mRNA levels (because we predicted that nonsense-mediated decay would make the mutated Otop1-/- mRNA unstable). However, the RNAseq traces show that all of the Otop1 mRNAs do have the deletion (Supplementary Figure 1). The 38-bp deletion preserves only the first 40 aa of OTOP1 and introduces a frame shift with a downstream stop codon. If translation starts at the next downstream methionine, it will produce a non-functional Otop1 protein due to deletion of the first two transmembrane domains (and reducing predicted OTOP1 protein from 66 kDa to 56 kDa).

We have added text to make this clearer (line 63-64) “*Otop1^-/-^* (*Otop1*^em1Lmn^ [7], from Dr. Emily Liman) mice have no detectable proton current in sour taste cells, in contrast to *Otop1^tlt/tlt^* mice which have a small, but detectable, residual proton current in sour taste cells [1].” (line 166-167) “The *Otop1^-/-^* mice are null for OTOP1 function [7] and exhibit a head tilt as seen in *Otop1^tkt/tlt^* mice [3], …”

RE OTOP1 Western: Please see the detailed response above to reviewer 1, Figure 1.

For the discrepancy between the Otop1-/- and Otop1tlt/tlt mice, we favor the hypothesis that there is no difference in their inflammatory and glucose physiology. One possibility is that the previous observations showing slightly increased insulin resistance and increased WAT inflammation in tlt mice are not reproducible and are due to stochastic variation. However, we have not performed a direct, side-by-side comparison of the two mouse strains, so to be both polite and complete we listed other possible explanations.

How the seahorse analysis of BAT mitochondrial function was exactly measured is confusing. The methodology mentioned collagenase digestion of BAT and filtered retained tissues were used for seahorse analysis. This is problematic, as no data showed that the retained tissue is the BAT. Collagenase digestion is expected to fractionate primary brown adipocytes from the Stromal vascular cell fraction. The retained tissues could be simply undigested fibrotic tissues. The cited reference (line 152, Refs 20 & 21) clearly used BAT explant without collagenase digestion.

The reviewer is concerned about the method used to obtain BAT samples for Seahorse analysis. We wanted to assay BAT without culturing to avoid any possible spurious effects. Much effort was put into validating the BAT tissue pieces assay; our apologies for not communicating this clearly in the original ms. We started with the method in ref [15] but found that the tissue gave only a small response to either FCCP or CL316243 stimulation. Thus, we optimized the method with mild collagenase digestion (not digesting to the single cell level) and mechanical trituration. The collagenase-digested pieces have the visual appearance of BAT (brownish and floating), an improved response to FCCP, and a very robust response to CL316243.

We have updated the Methods (section 2.2), giving more details and modified the Results (line 190-194): “The wild type BAT had the expected properties by Seahorse analysis, including a minimal effect of oligomycin indicating little ATP-linked respiration, a robust FCCP-induced maximally uncoupled oxygen consumption rate (OCR) of 210% of baseline, and a non-mitochondrial (antimycin- and rotenone-inhibited) OCR of ~30% of baseline (Figure 3A-C), all of which are comparable to studies of BAT adipocytes and improved over other tissue fragment assays [15, 22].”

Meanwhile, it is also puzzling why Palmitate did not increase basal respiration as compared to vehicle-treated one, since FFA is expected to activate FAO and uncoupling, increasing respiration. In addition, mitostress test is not a good way to assess glycolytic capacity. It is better to be measured by glycolysis stress test. Therefore, Figure 3 and 6 are questionable and needs to be clarified.

We believe that the pre-incubation with palmitate for 1 hour before the Seahorse analysis allowed the BAT to reach a new steady state, instead of showing an acute response to FFA.

As the reviewer notes, the mito-stress test is not designed to rigorously assess glycolytic capacity. Thus, we revised section 3.4, removing Figure 6C and only showing the change in OCR/ECAR ratio, reflecting as a shift in the aerobic-glycolytic balance.

The energy balance in males at 23°C does not add up together as these male Otop1-/- mice have higher food intake, lower physical activity, and higher RER without changes in TEE and body weights. Please explain.

Overall, the energy balance does add up. There was no difference in body weight at the time of the calorimetry experiment (27.1 g in WT vs 27.0 g in Otop1-/- mice; p=0.93). The food intake of the Otop1-/- mice (Figure 4H) was slightly greater during the dark phase (p=0.016) and slightly less during the light phase (p=0.098). Thus, when food intake is expressed per 24 hours, it is not different from the controls (p=0.13). We have modified the text to make this point clear (line 210-212) “*Otop1^-/-^* mice ate similar amounts per 24 h as controls, but with a greater percentage during the dark phase (71% in WT vs 79% in *Otop1^-/-^*, p=0.045).” The higher dark phase RER (Figure 4F) is consistent with the higher dark phase food intake.

The energy cost of physical activity has been studied in our lab (DOI 10.1016/j.molmet.2023.101699). When mice are studied at 23˚C, which is below thermoneutrality, the decreased heat production from decreased physical activity in Otop1-/- mice is replaced by increased cold-induced thermogenesis from brown fat. Thus, the lower physical activity level in Otop1-/- mice does not change the TEE or cause obesity.

What explains the sex differences in thermogenesis of Otop1-/- mice at different temperatures and after CL injection needs to be discussed.

There are known sex difference in mouse thermal physiology, for example, the higher body temperature in females. We added (lines 315-316) “There are known sex differences in mouse thermal physiology, including a higher Tb in females and a different response to fasting [36], with sex hormones contributing to the sex dimorphism [37].”

I’m not sure why RNA-seq data was only selectively presented in Fig. 8. How many overall DEGs were identified? Are there any pathways enriched? This information may give an overview of the physiological changes of Otop1-/- BAT. Furthermore, these data need to be confirmed by RT-PCR in BAT.

The RNA-seq data shows that there are not major changes in expression profile between Otop1-/- and controls. There were 921 DEGs observed (defined as: p<0.05 without multiplicity correction, fold change >1.5, and at least 1 sample with rpm >1). GO and pathway analysis identified response to cytokines, regulation of cytokine production, innate and adaptive immune response, and virus infection as the most affected pathways. As the reviewer suggested, we replicated the RNA-seq analysis using RT-PCR on a larger, independent set of BAT samples, focusing on the most changed genes. Unfortunately, since the most changed RNA-seq DEGs did not replicate, we do not report detailed analysis of the RNA-seq dataset.

Not sure how similar insulin sensitivity resulted in improved glucose tolerance in HFD-fed Otop1-/- mice (Supplemental Figure 4K&M).

Supplementary Figure 4K showed ITT results for female WT and Otop1-/- mice under HFD, with no significant difference in insulin sensitivity. Supplementary Figure 4M showed improved glucose tolerance in GTT for female WT and Otop1-/- mice under HFD, as GTT AUC was presented in Supplementary Figure 4N.

Insulin tolerance test is normally performed after a short (4-6h) fast instead of under nonfasting conditions.

We previously compared nonfasting vs 4h fasting conditions for the ITT and found more consistent results with nonfasting and have used and published this method since (DOI: 10.1016/j.molmet.2020.02.012).

Discussion on Supplemental Figure 6 is too much speculation. Current data provides no such insights.

As suggested, we have revised and condensed the discussion and removed the associated supplementary figure.

Please indicate at what age the mice were started on HFD in Materials and Methods.

We indicate age in figure legends. In fig 2 legends, the mice were started with HFD at the age of 30 weeks for male and 26 weeks for female.

Please indicate what software was used to perform statistic analyses.

We added to Methods (line 154) “Statistical analyses were done using Prism (version 9.0; GraphPad Software, Inc.).” and added details to the figure legends.

Reviewer #3: The manuscript by Tu et al. reported the function of OTOP1 in BAT, where OTOP1 expression level was enriched, using OTOP1 deficient mice. Although HFD-induced body weight gain was comparable, fasting-induced energy expenditure was reduced in OTOP1-deficient mice. Also, the oxygen consumption rate in BAT from OTOP1 KO mice was diminished upon FCCP treatment, whereas it was enhanced upon CL treatment.
Unfortunately, the purpose and interpretation of each figure are unclear. Thus, it is hard to understand the physiological roles of OTOP1. Moreover, provided data are not sufficient to suggest BAT is the main organ mediating those metabolic changes in OTOP1-deficient mice. Furthermore, it is likely that the authors should collect more data showing that OTOP1-mediated metabolic change is mediated through its proton channel function. Followings are specific comments to improve the study.

1. OTOP1 BAT showed different results on OCR, which was reduced upon FCCP (Figure 3A) but elevated upon CL treatment (Figure 6A). Also, palmitate addition showed different effects on the OCR in each experiment. The authors described that these results could be associated with the influx or efflux of protons depending on cellular pH (text lines 265-279). However, the authors did not provide the data showing proton flux. Moreover, it is possible that palmitate could act as an energy source or lipotoxic mediator rather than a proton carrier. Thus, for the author’s proposed model, they need show intracellular pH or proton flux to elucidate the roles of OTOP1 and palmitate in those experiments.

In response to the reviewer’s comments, we have revised and condensed the discussion and removed the associated supplementary figure. We have included references that support the ideas discussed. We are not set up to do pH imaging in brown adipocytes and believe that it is beyond the scope of this manuscript.

2. In Figure 3, the authors described that ‘these data show that Otop1 loss reduces maximal, but not basal respiration, in contrast to the effect of Ucp1 loss which reduces basal respiration’ (text lines 171-174). However, basal respiration cannot be compared because WT is not included in Supplementary Figure 2. Thus, it needs to show the OCR of BATs from all four groups (WT vs. OTOP1 KO vs. UCP1 KO vs. OTOP1/UCP1 DKO) in one graph to compare basal and FCCP-induced OCR.

As the reviewer suggested, we now show all four genotypes in a revised Figure 3A-D. Thus, the data presented now illustrates and supports the description in the text (lines 201-206).

3. Related to Figures 4 and 5, it needs to explain the rationale for the author’s hypothesis (text p.5, lines 178-180) regarding the thermal physiology experiments. Also, the authors should describe the aims of this experimental design (23 ℃ -> fasting -> 8 ℃ -> 35 ℃).

The manuscript has been edited to better explain the purpose. We examined the thermal physiology of Otop1-/- mice as a screen for in vivo effects, since BAT is crucial for defending Tb. We exposed the mice to different stresses, such as fasting, cold, and hot, to see how they responded (lines 212, 216). “We next examined the thermal physiology of *Otop1^-/-^* mice as a *in vivo* screen of BAT function. … These results rule out major defects in BAT function.”

4. One of the consistent changes in metabolic phenotypes is the reduced Tb and TEE in OTOP1 KO mice (Figures 4K-L, Figures 5K-L). The authors should investigate the roles of BAT OTOP1 in the fasting condition. The comparison of histology or thermogenic gene expression in BATs from control and OTOP1 KO mice upon fasting might be informative to reveal the function of BAT OTOP1-mediated thermogenesis or energy expenditure.

As suggested, we studied BAT gene expression in WT and Otop1-/- mice fasted for 24h (new Supplementary Figure 3). There were greater increases in *Cidea*, *Pnpla2* (ATGL), and *Slc27a2* (FATP2) expression in the *Otop1^-/-^* mice (p values >0.01 and <0.05 without multiplicity correction).

5. In Figure 6, the authors have to show the CL responses (lipid droplet size by histology and metabolic gene expression levels, including thermogenic genes) in BATs from WT and OTOP1 KO mice to examine the roles of BAT OTOP1 in CL-mediated energy expenditure

We added text to make clear that what we are studying with this single dose of CL316243 is an acute response that is a good test of maximal BAT capacity (examples using this are: DOI: 10.1172/JCI8437 and DOI: 10.1074/jbc.M114.568204). Mice housed at room temperature are already cold stressed, with their BAT under constant sympathetic stimulation.
