# Supplementary material for: Loss of Otopetrin 1 affects thermoregulation during fasting in mice
Source: PLoS One. 2023 Oct 9;18(10):e0292610. doi: 10.1371/journal.pone.0292610 (PMC10561838; doi:10.1371/journal.pone.0292610)
Supplement: Attachment — Submitted filename: response to reviewers 2023.08.28.docx [file pone.0292610.s011.docx]

**RE:** Manuscript Number: PONE-D-22-34443R1

**Title:** " Loss of Otopetrin 1 affects thermoregulation during fasting in mice"

August 28, 2023

Dear Dr Qiao and reviewers,

We thank you and reviewer 3 for the comments and invitation to submit a further revised manuscript. As detailed below we have addressed the issue raised by reviewer 3 and believe the manuscript is now suitable for PLOS One.

Regards,

Marc

Response to Reviewers:

Reviewer #3: The authors have made efforts to address the reviewer’s comments in revised manuscript. However, the revised manuscript does not clearly demonstrate this reviewer’s suggestions. In particular, the authors describe the role of OTOP1 in fasting-induced hypothermia and increased CL-mediated thermogenesis in OTOP1 KO mice in the discussion section (lines 291-305), but there is a lack of experimental evidence supporting their proposed model. Thus, it is difficult to accept the conclusions without any experimental evidence. To strengthen the proposed model, the authors have to compare the intracellular pH between WT and OTOP1 deficient brown adipocyte in response to FCCP and beta3 adrenergic agonist. This can be accomplished by utilizing appropriate dyes or a pH-sensitive indicator such as pHluorin (pH-sensitive indicator pHluorin, reference DOI: https://doi.org/10.7554/eLife.77946). This data would provide direct evidence for the involvement of OTOP1 in modulating intracellular pH under fasting or CL conditions.

*The reviewer requested experimental support for the model proposed in the Discussion of the revised manuscript. As suggested by the reviewer, we set up a fluorescence assay to measure intracellular pH using the dye BCECF. This work is new to our lab and, to summarize many experiments, we have not been able to reproduce the expected alkalinization of intracellular pH upon adrenergic stimulation (doi: 10.1152/ajpcell.1994.267.2.C349) with CL316243 and acidification with FCCP treatment (doi: 10.1152/ajpheart.00932.2012). Thus, to satisfy the reviewer’s suggestion, we have completely removed the model from the discussion—lines 291-305 become lines 291-294. We believe that these changes satisfy the concerns of the reviewer and editor.*

*Original:*

*“**Ex vivo, Otop1^-/-^ BAT basal mitochondrial respiration was unchanged, while maximal respiration was reduced and β3-adrenergic stimulated respiration was increased when compared to controls. Basally, taking into account the intracellular pH, extracellular pH, and plasma membrane potential, there is a small electrochemical gradient across the brown adipocyte plasma membrane that predicts inward flux of protons via OTOP1 [30, 31]. In the presence of FCCP, the plasma membrane is depolarized and the cytosol is acidified [32]; under these conditions OTOP1 could instead mediate proton efflux, thereby increasing intracellular pH. With FCCP, the lack of OTOP1 function might decrease the intracellular pH, potentially inhibiting processes including CPT1-mediated fatty acid transport [33] and glycolysis [34], thereby leading to the observed reduction in Otop1^-/-^ OCR.*

*Under stimulated conditions, such as with a β3-adrenergic agonist, BAT cytosol is alkalinized [31]. Cytoplasmic alkalinization could increase OTOP1-mediated proton influx across the plasma membrane. Thus, loss of Otop1 would reduce that pH gradient across the inner mitochondrial membrane and thus cause a compensatory increase mitochondrial respiration to maintain the pH gradient. Additional palmitate could compensate for the loss of Otop1, as its protonated form might act as carrier to increase proton influx.”*

*Revised:*

*“Ex vivo, Otop1^-/-^ BAT basal mitochondrial respiration was unchanged. In contrast, maximal respiration was reduced and β3-adrenergic stimulated respiration was increased when compared to controls. Further studies are required to understand the mechanistic basis underlying how the loss of OTOP1 function causes these changes.”*
